# Impact of Smoking on the Efficacy of Human Autologous Oral Mucosal Epithelial Cell Sheet Transplantation for Treating Limbal Stem Cell Deficiency

**DOI:** 10.1155/sci/3769266

**Published:** 2026-01-05

**Authors:** Mingqi Zhang, Yuqiang Zheng, Tao Yao, Le Wang, Hui Yu, Zhuoshi Wang

**Affiliations:** ^1^ Department of Stem Cell Center of Precision Medicine Innovation Institute, He University, No. 66 Sishui Street, Hunnan District, Shenyang, 110163, Liaoning Province, China, he-edu.com; ^2^ Liaoning Key Lab of Ophthalmic Stem Cells, He University, Shenyang, Liaoning Province, China, he-edu.com; ^3^ He Eye Specialist Hospital, Shenyang, Liaoning Province, China; ^4^ School of Life Science, Liaoning University, Shenyang, Liaoning Province, China, lnu.edu.cn

**Keywords:** acrolein, autologous oral mucosal epithelial cells, limbal stem cell deficiency (LSCD), smoking effects, therapeutic efficacy

## Abstract

Limbal stem cell deficiency (LSCD) results from the loss or dysfunction of limbal stem cells, posing a significant challenge due to limited treatment options. Autologous oral mucosal epithelial cell (OMEC) sheet transplantation is an innovative therapy, but its effectiveness in smokers remains unclear. This study aims to investigate the impact of smoking on the efficacy of autologous OMEC sheet transplantation for LSCD and explores how acrolein, a major cigarette smoke component, affects the biological properties of these cells. This retrospective cohort study included 13 LSCD patients (13 eyes), divided into never smokers (seven eyes) and smokers (six eyes), all of whom received autologous OMEC sheet transplantation. The study compared colony‐forming abilities and sheet thickness between the groups, assessed corneal epithelial repair postoperatively, and conducted in vitro experiments treating human OMECs (hOMECs) with acrolein. Evaluations focused on colony‐forming ability, stem cell marker protein P63 expression, and the secretion of repair factors (transforming growth factor‐beta [TGF‐β], basic fibroblast growth factor [bFGF], and hepatocyte growth factor [HGF]) as well as the wound healing potential in human corneal epithelial cells. Postoperative results showed significant improvements in corneal epithelial defects, neovascularization, and best visual acuity in never smokers. However, adhesions recurred in both groups, with earlier recurrence in smokers. In vitro, acrolein significantly inhibited the proliferation of OMECs, reduced P63 expression, and decreased the secretion of TGF‐β and HGF, though bFGF levels remained unchanged, impairing wound healing in scratched corneal epithelial cells. Smoking adversely affects the efficacy of autologous OMEC transplantation for LSCD, with acrolein as a potential key factor. Future research should focus on understanding the mechanisms by which smoking impacts OMECs and developing improved therapeutic strategies for smokers with LSCD.

**Trial Registration:** ClinicalTrials.gov identifier: NCT03015779

## 1. Introduction

The cornea of the eye provides most of the refractive power, and its clarity is vital for good vision. Stem cells from the palisades of Vogt in the limbal musculature are being increasingly used for corneal repair and maintenance [1]. Defects in limbal epithelial cells (limbal stem cell deficiency [LSCD]) lead to persistent corneal inflammation, corneal surface conjunctivalization, corneal neovascularization, and corneal defects recurring overtime [2, 3].

### 1.1. Autologous and Allogeneic Limbal Epithelial Transplantation (auto‐ and allo‐LET)

LET remains the mainstay of treatment for LSCD, aiming to restore the corneal epithelial stem cell niche.

• auto‐LET uses tissue harvested from the patient’s healthy eye. This approach eliminates the risk of immune rejection and avoids the need for systemic immunosuppression [4]. However, it carries significant drawbacks, including the risk of inducing iatrogenic LSCD in the donor eye and its inapplicability to bilateral LSCD [5, 6].
• allo‐LET, derived from cadaveric or living‐related donors, expands the donor pool and can be used for bilateral disease. Nevertheless, it is associated with immune rejection and necessitates long‐term systemic immunosuppression, which may lead to secondary complications and graft failure [7, 8]. Reported success rates for allogeneic transplantation vary widely, typically ranging between 40% and 70%, depending on donor source, immune status, and postoperative management [9, 10].

### 1.2. Emerging Alternatives: Cultivated LET (CLET) and Simple LET (SLET)

To overcome these limitations, several advanced surgical approaches have been developed.

CLET allows ex vivo expansion of a small limbal biopsy on biological or synthetic scaffolds, reducing donor site morbidity [11].

SLET further simplifies the process by directly placing small limbal explants on an amniotic membrane (AM) on the recipient cornea, enabling in vivo cell expansion with promising clinical outcomes [12].

Despite these innovations, the need for healthy limbal tissue still limits their application in bilateral LSCD.

### 1.3. Alternative Cell Sources

As a result, researchers have investigated alternative autologous cell sources for ocular surface reconstruction. These source, based on their origin, include embryonic stem cells and adult stem cells [13, 14]. Of these, autologous oral mucosal epithelial cells (OMECs) have been proposed as the appropriated candidate due to their similar morphological and cytochemical features [15, 16]. The use of cultured oral mucosa epithelial cells for ocular surface regeneration has been around for almost a decade. Tissue‐engineered oral mucosal cell sheet has been successfully explored to treat various causes of ocular trauma and congenital esophageal atresia, its safety and efficacy has been been widely verified [17, 18].

The oral cavity acts as the body’s entrance for nutriments, pollutants, and microorganisms. It is possible for harmful substances to accumulate in the oral mucosa and lead to OMECs damage [19, 20]. Cigarette smoke has been shown to increase the risk of oral mucosa dysplasia, leukoplakia, and carcinoma [21]. OMECs for clinical therapeutic use are sourced from oral mucosa biopsies and the quality of oral biopsy is closely related to OMECs treatment outcomes. However, few studies regarding the correlation between the quality of biopsy and the therapeutic outcomes of applying OMECs to treat LSCD are reported. Acrolein, known to be one of the main components of cigarette smoke, is highly soluble in water and alcohol and can cross the membranes by passive diffusion [22]. Previous studies have demonstrated that acrolein have given rise to atherosclerosis [23], cardiovascular disease [24], pulmonary inflammation [25], and retinal disease [26] and affected the defense function of the oral mucosal cells [27]. However, the regenerative potentials effects of acrolein on OMECs and repair functions in the cornea are still unknown.

Therefore, the present study aimed to investigate the impact of smoking on the clinical efficacy of human OMECs (hOMECs) in LSCD patients and to explore the potential mechanisms by which acrolein influences the biological properties of OMECs.

## 2. Materials and Methods

### 2.1. Study Design and Patients

This was a retrospective cohort study including patients with LSCD who underwent cultured autologous OMEC sheet (CAOMECS) transplant between 2017 and 2020. The clinical trial was approved by the Institutional Review Board of He Eye Specialist Hospital. Institutional consent forms, including consent for future publication, were signed by all patients.

We included 13 male patients (13 eyes) with unilateral LSCD caused by ocular chemical injuries or thermal burns, who were hospitalized and underwent CAOMECS transplant at our hospital. Patients were divided into two groups based on their smoking history, with six patients (six eyes) in the smoking group and seven patients (seven eyes) in the nonsmoking group. Smoking history was obtained by patient self‐report, including duration and average number of cigarettes per day.

Eligible patients had total LSCD for at least 6 months following ocular chemical injury or thermal burn and had not responded to prior conservative management, such as medical therapy and AM transplantation. None of the patients had undergone prior corneal transplantation, as corneal grafting is typically reserved for visual rehabilitation after successful ocular surface reconstruction. In cases of LSCD secondary to chemical burns, corneal transplantation before surface reconstruction is only considered for tectonic purposes when corneal integrity is threatened [28]. Diagnostic criteria included complete disappearance of the palisades of Vogt in the limbal area, pathological corneal epithelium, persistent corneal defects, corneal neovascularization, and conjunctival epithelial cells, with or without symblepharon. All patients were older than 18 years of age.

Exclusion criteria included systemic infection, oral injuries, a history of hypersensitivity or allergy to antibodies or serum, malignant tumors, and pregnancy or lactation.

### 2.2. Preparation and Use of Autologous Serum

Autologous serum was obtained from each patient’s peripheral blood collected 1 week prior to surgery. Whole blood (~20 mL) was allowed to clot at room temperature for 1 h and then centrifuged at 3000 rpm for 10 min. The supernatant serum was filtered through a 0.22 µm filter and aliquoted for use as a supplement in the culture medium (5% *v*/*v*) and as postoperative eye drops (diluted to 20% with sterile saline). The autologous serum eye drops were instilled every 2 h during the first postoperative week and then gradually tapered based on epithelial healing status.

### 2.3. Cell Sheet Fabrication and Quality Control

Human oral mucosal tissue was obtained in accordance with the tenets of the Declaration of Helsinki for research. The epithelial cell sheets were prepared using optimized previously reported methods. In brief, a 2–3 mm^2^ buccal mucosal tissue sample was harvested from the patient’s cheek and placed into a 50 mL centrifuge tube containing Dulbecco’s modified eagle’s medium (DMEM; Sigma–Aldrich, MO, USA) supplemented with 100 µg/mL streptomycin (Meiji Seika Pharma, Tokyo, Japan) and 1.0 µg/mL amphotericin B (Fungizone, Bristol‐Myers Squibb, NY, USA). The tissue was treated with 10 mg/mL dispase (Invitrogen, CA, USA) for 2 h at 37°C and 2.5 mg/mL trypsin (Thermo Fisher Scientific, MA, USA) for 20 min at 37°C to isolate OMECs. The disaggregated OMECs were suspended in an oral mucosal epithelial culture medium (OECM) composed of a basal medium of DMEM and Ham’s F‐12 mixture, supplemented with 10% fetal calf serum, 0.12 U/mL insulin (Humulin; Eli Lilly, IN, USA), 10 ng/mL EGF, 0.4 µg/mL isoproterenol, 40 µg/ml gentamicin (Gentacin, Takata Pharmaceutical, Saitama, Japan), 0.25 µg/mL amphotericin B, and 5% autologous serum. The OMECs (2 × 10^4^ cells/cm^2^) were plated in a six‐well plate containing mitomycin C (MMC)–inactivated human foreskin‐derived fibroblast cells as a feeder layer and cultured in OECM at 37°C under 5% CO_2_. The culture medium was replaced every 48 h, and the cells reached confluence to form a continuous epithelial sheet after approximately 21 days of culture.

Before transplantation, the cell sheets were detached by treatment with 5 mg/mL dispase for 10 min, rinsed three times with 0.09% NaCl, and transferred to a PVDF ring.

The quality control of the cell sheets was conducted according to previously reported methods [17]. Culture media collected after media replacement were subjected to sterility tests, including (1) cultivation for aerobic and anaerobic bacteria and fungi using a microbial detection system (BacT/Alert), (2) endotoxin detection using a limulus amoebocyte lysate assay, and (3) mycoplasma testing by cultivation and PCR detection of DNA derived from *Mycoplasma pneumoniae*.

The clonogenic potential and histological structure of at least one cell sheet were analyzed. The colony‐forming efficiency (CFE) was calculated using the formula: CFE = (number of colonies/number of cells seeded) × 100%. A total of 500 cells were plated on MMC–inactivated human fibroblast cells in 25 cm^2^ flasks. After 14 days of cultivation, the flasks were carefully scored for the presence of colonies, which were stained with 1% rhodamine B (Sigma, St. Louis, Missouri, USA) and classified by clonal types. Colonies were photographed using an Olympus microscope.

For histological assessment, cell sheets were fixed in 10% neutral‐buffered formalin solution and embedded in paraffin to prepare 8‐µm sections. The paraffin‐embedded sections were stained with hematoxylin and eosin (H&E) and observed under an optical microscope (Olympus BX51).

### 2.4. Surgical Techniques and Evaluation of Trial Outcomes

The cell sheet was performed only after the ocular surface had remained clinically quiet for at least 6 months following chemical or thermal injury. This waiting period ensured that ocular surface inflammation had fully subsided, as subtle subclinical inflammation may persist despite an apparently stable surface. The timing was determined in accordance with previous recommendations for ocular surface reconstruction procedures [29]. The surgical procedure adhered to previously established protocols [30]. In brief, all transplants were conducted under peribulbar anesthesia by a single experienced surgeon. The procedure began with the careful removal of the fibrovascular pannus on the cornea and the subconjunctival scar tissue to expose the corneal stroma. Next, the CAOMECS was placed on the bare corneal stromal bed with the epithelial cells facing upward. This was immediately covered with a rehydrated AM (JiXi RuiJi BioTechnology Co., Ltd, Jiangxi, China), which was sutured at the conjunctival edge using interrupted polyglactin 910 sutures (Coated Vicryl; Ethicon, Tokyo, Japan).

Postoperatively, patients received topical antibiotics and corticosteroids, initially administered four times daily, then reduced to three times daily. Additionally, 0.3% preservative‐free hyaluronic acid (Hyalein‐Mini; Santen Pharmaceutical Co.) and 20% autologous serum eye drops were used to aid epithelial healing. After 1 month, the medication regimen was gradually tapered based on the patient’s condition, with sodium hyaluronate treatment continued for up to 6 months to support ongoing recovery.

Evaluation endpoints were assessed at the initial baseline visit and at 6 months posttransplantation. The primary endpoints included persistent epithelial defect, neovascularization, symblepharon, best visual acuity, and patient‐reported functional symptoms such as photophobia, watering, and pain. These assessments were conducted using slit lamp examination by the same ophthalmologist to ensure consistency.

Success in the study was defined by either improvement or stabilization across all measured criteria. Conversely, if any criterion exhibited deterioration, the outcome was considered a failure [17]. The specific criteria for assessing the success or failure of the treatment included:

• Persistent epithelial defect: The severity of the persistent epithelial defect was graded on a scale:
  No ulcer: Assigned a score of 0.
  Mild: Characterized by an ulcer with a diameter of less than 5 mm, assigned a score of 1.
  Moderate: Defined as an ulcer with a diameter between 5 and 7 mm, assigned a score of 2.
  Severe: Identified by an ulcer with a diameter greater than 7 mm, assigned a score of 3.
• Neovascularization: The presence and severity of new blood vessel growth in the cornea were graded as follows:
  Absent: No evidence of angiogenesis (no visible vessels).
  Mild: Low angiogenic activity (fewer than three visible vessels).
  Moderate: Moderate angiogenic activity (three to eight visible vessels).
  Severe: High angiogenic activity (more than eight visible vessels and/or the corneal tissue between arterioles contained anastomosing microvessels).
• Symblepharon: The degree of adhesion between the eyelid and the eyeball was categorized as:
  Absent: Assigned a score of 0.
  Mild: Assigned a score of 1; involving less than one‐third of the eyelid length or only the length of the palpebral conjunctiva.
  Moderate: Assigned a score of 2; involving more than one‐third but less than or equal to two‐thirds of the eyelid length, or the length of the palpebral conjunctiva but less than or equal to the length of the tarsus.
  Severe: Assigned a score of 3; involving more than two‐thirds of the eyelid length or close to total ankyloblepharon.
• Visual acuity: Visual acuity outcomes were converted into logarithm of the minimum angle of resolution (logMAR) scores for comparative analysis.
• Photophobia scale: No complaint, fear of luminosity corrected by sunglasses, frequent winking to light, inability to open eye.
• Watering scale: No complaint, dry, very dry, difficulty opening the eye.
• Pain scale: No complaint, occasionally, repetitive pain during the day, permanent.

### 2.5. Evaluation of Toxicity of Acrolein on hOMECs

Acrolein (purity > 95%) was provided by Prof. Xiangyu Cao from Liaoning University, China. Stock solutions were prepared by diluting acrolein in LC–MS‐grade water adjusted to pH 4.5, then aliquoted into 50 μL portions and stored at −80°C for up to 2 months. Treatment solutions were freshly prepared by diluting the stock solution in DPBS to concentrations ranging from 40 to 160 μM. Normal hOMECs, cryopreserved at passage 0, were purchased from Shanghai Qianxi Biotechnology Company. The cells were seeded at a density of 3 × 10^5^ cells per well in a six‐well plate coated with vitronectin (Gibco, Carlsbad, US) and maintained in OECM medium until they reached 90% confluence. Cells were treated with various concentrations of acrolein (0, 40, 80, 120, 160 μM) for 24 h. After incubation, cells were observed and photographed using an inverted microscope. Viable cells were counted posttrypsinization using trypan blue. Each condition was performed in triplicate and repeated four times (*n* = 4). IC_50_ calculations were conducted using online tools (https://www.aatbio.com/tools/ic50-calculator).

Cell viability was evaluated using the CCK‐8 assay. In brief, 2 × 10^3^ cells per well were seeded into 96‐well plates. After treatment with acrolein for 24, 48, and 72 h, cell viability was assessed according to the CCK‐8 assay instructions.

### 2.6. Evaluation of Acrolein on hOMEC Stemness

To assess the impact of acrolein on the stemness of hOMECs, cells at Passage 2 were cultured to 80% confluence and then treated with 80 μM acrolein for 24 h. The expression of the stem cell marker protein P63 was analyzed using semiquantitative immunofluorescence and real‐time RT‐PCR.

#### 2.6.1. Immunofluorescence Analysis

Cells were fixed with 4% paraformaldehyde for 30 min, washed, and permeabilized with 0.1% Triton X‐100 for 10 min. After blocking with 5% BSA for 30 min, cells were incubated with a primary antibody against P63 (abcam, ab32353, dilution 1:100) for 2 h at room temperature. Following three PBS washes, a fluorescent secondary antibody (abcam, ab150077, dilution 1:2000) was applied for 1 h at room temperature. Cells were then washed with PBS, mounted using DAPI, and photographed using an Olympus fluorescence microscope. ImageJ software was utilized for the analysis of P63‐expressing cells.

#### 2.6.2. RNA Extraction and Real‐Time RT‐PCR

Equal numbers of hOMECs were harvested after 24 h of acrolein exposure for total RNA extraction using the Trizol (invitrogen) method. Real‐time RT‐PCR was conducted to quantify the expression levels of P63. The primer sequences used were: forward primer for P63: F: GCAACGCCCTCACTCCTACAAC; reverse primer for P63: R: AGTCCATTCATGTCTCCAGCCATTG.

### 2.7. Condition Medium (CM) Collection and Secreted Factor Measurement

hOMECs, treated with 80 μM acrolein for 24 h or left untreated, were cultured in 75 cm^2^ flasks. Upon reaching confluence, the medium was replaced with 10 mL of DMEM/F12 medium per flask and incubated for 48 h. The supernatants were subsequently collected, filtered, and centrifuged at 2000 × *g* for 30 min to isolate the hOMECs‐CM. The hOMECs‐CM from both the acrolein‐treated and untreated groups were analyzed for their potential to promote corneal epithelial cell wound healing and were further evaluated for secreted protein levels using human enzyme‐linked immunosorbent assay (ELISA). The ELISA assays were conducted to quantify the concentrations of human transforming growth factor‐beta (TGF‐β), basic fibroblast growth factor (bFGF), and hepatocyte growth factor (HGF).

### 2.8. Human Corneal Epithelial Cell Wound Healing Assay

To evaluate the function of hOMECs‐CM, a human corneal epithelial cell line (obtained from Guangzhou, China) was seeded in 12‐well plates at a density of 1 × 10^5^ cells per well and incubated at 37°C with 5% CO_2_until a confluent monolayer was formed in DMEM/F12 medium containing 10% fetal bovine serum (FBS; Gibco). The confluent layer was scratched with a 100 μL sterile pipette tip after washing three times with DPBS. The culture medium was then replaced with hOMECs‐CM. Images (*n* = 3 per treatment) from the center of the wells were taken with an IX51 Olympus microscope equipped with a DPI 7.2 digital camera at time points 0, 24, and 48 h after scratching. The wound areas were analyzed and compared using ImageJ software.

### 2.9. Statistical Analysis

All statistical analyses were conducted using GraphPad Prism 9.0. A *p*‐value of less than 0.05 was considered statistically significant. The preoperative characteristics of patients were compared using Student’s *t*‐test, except for the cause of the condition, which was analyzed using Fisher’s exact test. Kaplan–Meier and log‐rank tests were employed to compare the probability of survival between the two groups. Pearson correlation analysis was used to assess the impact of age.

To compare pre‐ and posttreatment grading scores between the smoker and never smoker groups, two‐way analysis of variance (ANOVA) was performed. For in vitro cell experiments, data were presented as mean ± standard deviation (SD). One‐way ANOVA followed by Bonferroni post hoc multiple comparison test was utilized to identify significant differences (*p*  < 0.05) between treatment groups. All experiments were conducted in triplicate and repeated at least three times to ensure reliability.

## 3. Results

### 3.1. Evaluation of CFE and Cell Sheet Thickness

Clonal formation assays revealed that OMECs from never smokers formed significantly more colonies compared to those from smokers (never smokers: CFE = 2.25% ± 0.26% vs. smokers: 0.97% ± 0.49%, *p* < 0.01; Figure 1A). The thickness of the OMEC sheets also differed significantly between the groups, with never smokers showing greater sheet thickness (50.94 ± 8.47 μm; Figure 1B1) compared to smokers (33.88 ± 10.64 μm, Figure 1B2; *p* < 0.05, Figure 1B3). Further analysis indicated that age did not significantly influence the number of stem cell colonies or the viability of cells (Figure 1C).

Figure 1Impact of smoking on colony formation and sheet thickness. (A) Comparison of colony‐forming efficiency (CFE) between smokers and never smokers. Subpart (A1) shows higher colony counts in never smokers, while Subpart (A2) represents colony status in smokers, with significant differences indicated by statistical analysis (A3). (B) Representative images and statistical comparison of epithelial sheet thickness in smokers and nonsmokers. Subpart (B1) shows thicker and denser sheets in never smokers, while smokers have thinner sheets with larger cells (B2). (B3) Statistical comparison of epithelial sheet thickness between smokers and nonsmokers. Data are presented as mean ± SD. (C) Correlation analysis between age, CFE (C1), and number of viable cells (C2). Results show no significant age‐related differences.  ^∗^ indicates *p*  < 0.05;  ^∗∗^ indicates *p*  < 0.01.

**Figure:**
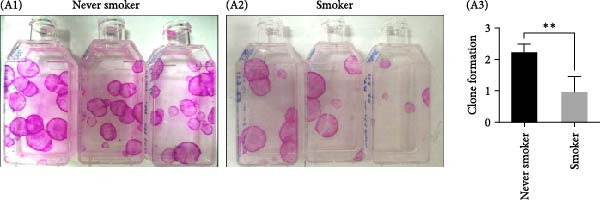
(A)

**Figure:**
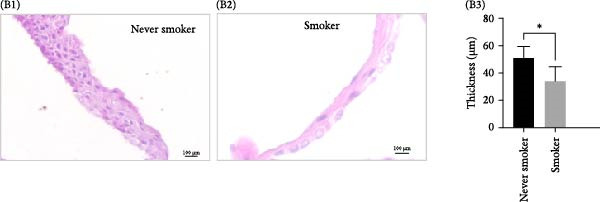
(B)

**Figure:**
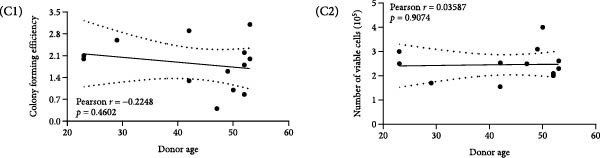
(C)

### 3.2. Evaluation of Posttreatment Changes Between Smoker and Never Smoker Groups

The baseline characteristics of patients in the smoker and never smoker groups were comparable before treatment (Table 1). After undergoing autologous OMEC sheet transplantation, both groups demonstrated significant improvements in corneal epithelial defects and a reduction in neovascularization. Initially, symblepharon, or adhesion between the eyelid and eyeball, was present in six patients from the smoker group and three patients from the never smoker group (Figure 2C,D). Posttreatment, improvements were observed in three patients from the smoker group and one patient from the never smoker group. However, the smoker group experienced earlier recurrences of symblepharon (Figure 2A). Visual acuity improved in nine patients overall, with two from the smoker group and seven from the never smoker group reporting enhanced vision (Figure 2B). Both groups also showed reductions in symptoms such as photophobia, watering, and pain (Table 2). The treatment was considered effective, achieving stable or improved outcomes in five eyes (71.4%) in the never smoker group and in two eyes (33.3%) in the smoker group during the 6‐month follow‐up. The primary cause of treatment failure in both groups was the recurrence of the condition.

Figure 2Changes in corneal evaluation parameters before and after treatment. (A) Recurrent symblepharon rates posttreatment. (B) Comparison of best‐corrected visual acuity pre‐ and posttreatment between groups. (C) Changes in corneal evaluation parameters in nonsmoker patients pre‐ and posttreatment. (D) Changes in corneal evaluation parameters in smoker patients pre‐ and posttreatment.  ^∗^indicates *p* < 0.05.

**Figure:**
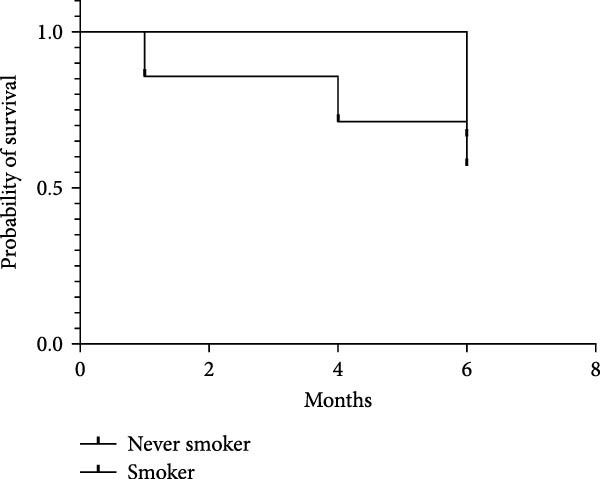
(A)

**Figure:**
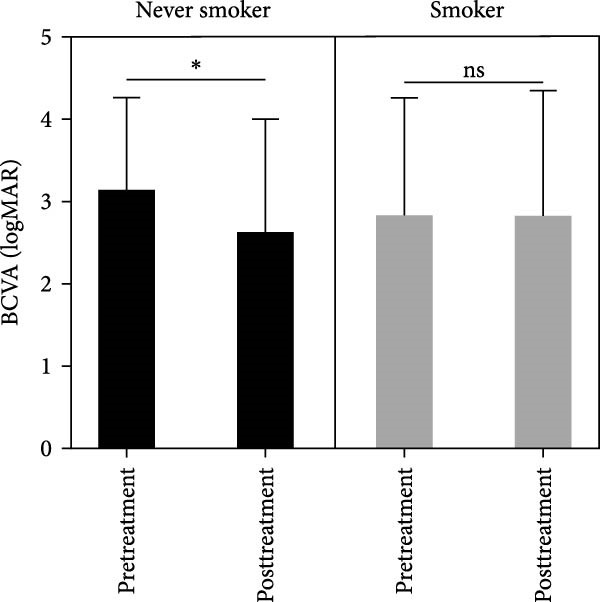
(B)

**Figure:**
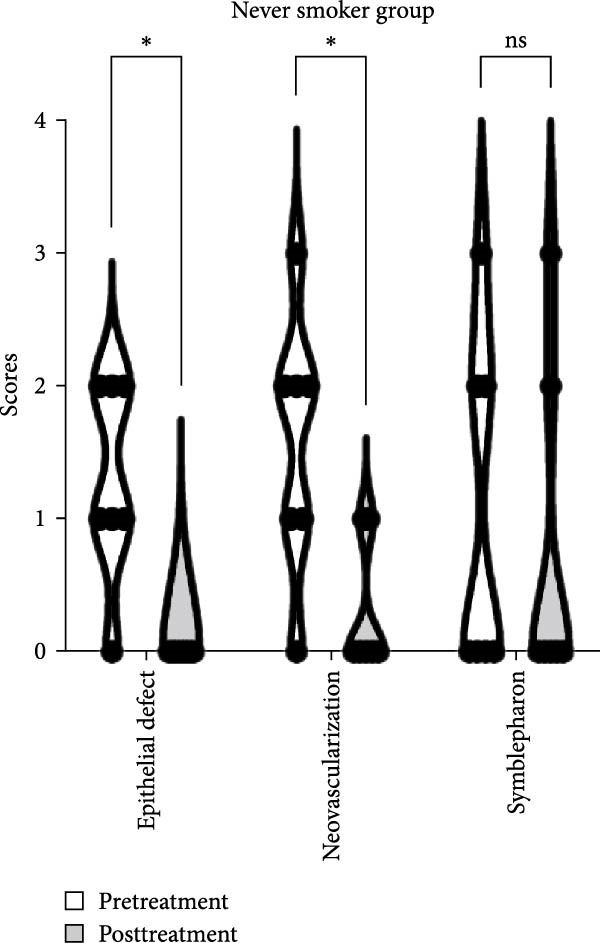
(C)

**Figure:**
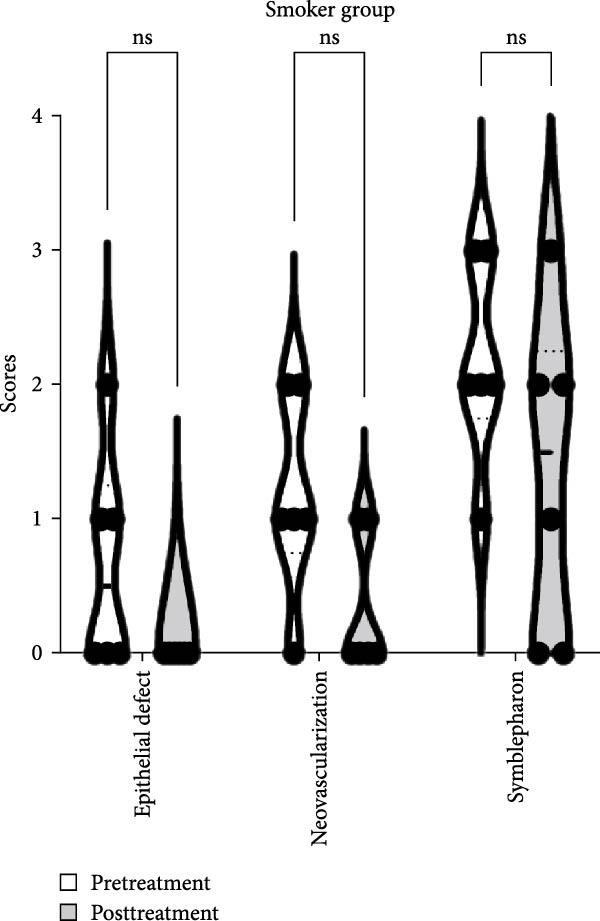
(D)

**Table 1:** Preoperative characteristics of patients with LSCD.

Characteristics	Never smoker	Smoker	*p*
Number of eyes	7	6	—
Age (years)	—	—	0.0942
Mean (SD)	38 (13.93)	48.83 (3.97)	—
Range	23–53	42–53	—
Causes	—	—	0.5594
Chemical burn	1	2	—
Thermal burn	6	4	—
Ocular status scores (median)			
Persistent epithelial defect	1	1.5	0.5902
Neovascularization	2	1	0.4267
Symblepharon	0	2	0.0782

**Table 2:** Details of the evaluation items for the 13 patients at inclusion and 180 days.

Characteristics						Preoperative/postoperative (6 M) of ocular events							
Number	Age	Sex	Eye	Initial disease	Smokers	Epithelial defect	Neovascularization	Symblepharon	Photophobia	Watering	Pain	Visual acuity	Efficacy
1	52	M	R	Chemical injuries	No	Moderate/no ulcer	Severe/mild	Absent/steady	Frequent winking to light/no complaint	Very dry/no complaint	Occasionally/no complaint	LP/HM50	Improve
2	52	M	R	Thermal burns	No	Mild/no ulcer	Moderate/absent	Absent/steady	Fear of luminosity corrected by sunglasses/no complaint	Dry/no complaint	Occasionally/no complaint	FC10/0.05	Improve
3	23	M	L	Thermal burns	No	Moderate/no ulcer	Moderate/mild	Moderate/absent	Fear of luminosity corrected by sunglasses/no complaint	No complaint/steady	No complaint/steady	LP/HM50	Improve
4	23	M	L	Thermal burns	No	No ulcer/steady	Absent/steady	Severe/recurrence	No complaint/steady	Dry/no complaint	Repetitive pain during the day/no complaint	0.1/0.6	Worsen
5	29	M	R	Thermal burns	No	Mild/no ulcer	Mild/absent	Moderate/recurrence	Frequent winking to light/no complaint	Dry/no complaint	Occasionally/no complaint	FC20/FC50	Worsen
6	53	M	R	Thermal burns	No	Moderate/no ulcer	Mild/absent	Absent/steady	Fear of luminosity corrected by sunglasses/no complaint	Dry/no complaint	No complaint/steady	LP/HM50	Improve
7	34	M	R	Thermal burns	No	Mild/no ulcer	Moderate/ absent	Absent/steady	No complaint/steady	Dry/no complaint	Occasionally/no complaint	HM 20/HM100	Improve
8	49	M	L	Thermal burns	Yes	No ulcer/steady	Absent/steady	Severe/mild	No complaint/steady	Dry/no complaint	Occasionally/no complaint	HM20/LP	Worsen
9	50	M	L	Thermal burns	Yes	Mild/no ulcer	Mild/mild	Severe/recurrence	Frequent winking to light/no complaint	No complaint/steady	Occasionally/no complaint	HM 50/HM50	Worsen
10	53	M	L	Chemical injuries	Yes	Moderate/no ulcer	Moderate/mild	Mild/absent	No complaint/steady	Dry/no complaint	Occasionally/no complaint	0.1/0.2	Improve
11	47	M	R	Thermal burns	Yes	Mild/no ulcer	Mild/absent	Moderate/recurrence	No complaint/steady	No complaint/steady	No complaint/steady	0.1/0.1	Worsen
12	52	M	R	Thermal burns	Yes	No ulcer/steady	Mild/absent	Moderate/recurrence	Frequent winking to light/no complaint	Dry/no complaint	No complaint/steady	LP/LP	Worsen
13	42	M	L	Chemical injuries	Yes	No ulcer/steady	Moderate/ absent	Moderate/absent	No complaint/steady	Dry/no complaint	Occasionally/no complaint	HM20/HM50	Improve

These findings suggest that while autologous OMEC sheet transplantation offers clinical benefits for LSCD patients, smoking is associated with earlier recurrences and potentially less pronounced improvements.

### 3.3. Effect of Acrolein on Cell Viability

We further evaluated the impact of acrolein, a major component of cigarette smoke, on the viability of hOMECs. The CCK‐8 assay was used to assess cell viability after treatment with varying concentrations of acrolein over a 24‐h period. The results indicated that at a concentration of 80 μM acrolein reduced cell viability to 41% (Figure 3A,B). As the concentration of acrolein increased, cell viability progressively decreased. The half‐maximal inhibitory concentration (IC_50_) was calculated to be 80 μM, indicating that this concentration significantly impairs cell viability. Consequently, subsequent experiments utilized 80 μM acrolein for further analysis. Notably, treatment with 80 μM acrolein led to significant cell death after 24, 48, and 72 h (Figure 3C).

Figure 3Effect of acrolein on the viability of human oral mucosal epithelial cells. (A (A1–A5)) Photographs showing the impact of different concentrations of acrolein on human oral mucosal epithelial cells. (B) Relationship between acrolein concentration and cell viability. (C) Effect of 80 µM acrolein on cell proliferation at various time points.  ^∗∗^ indicates *p* < 0.01;  ^∗∗^^∗∗^indicates *p*  < 0.0001.

**Figure:**
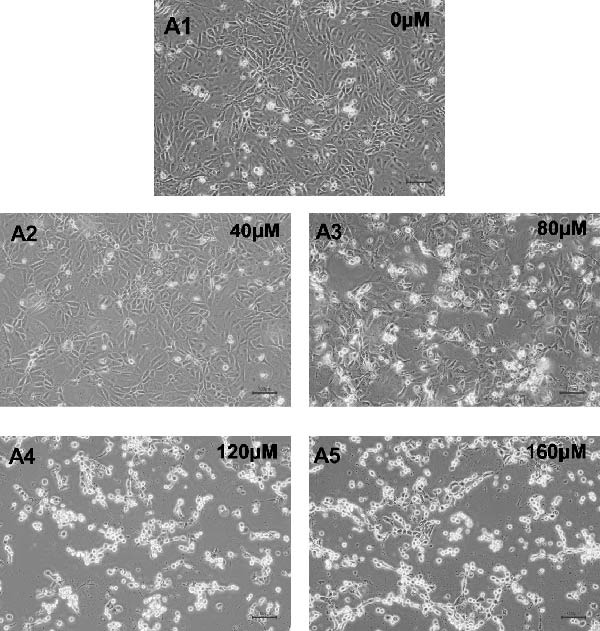
(A)

**Figure:**
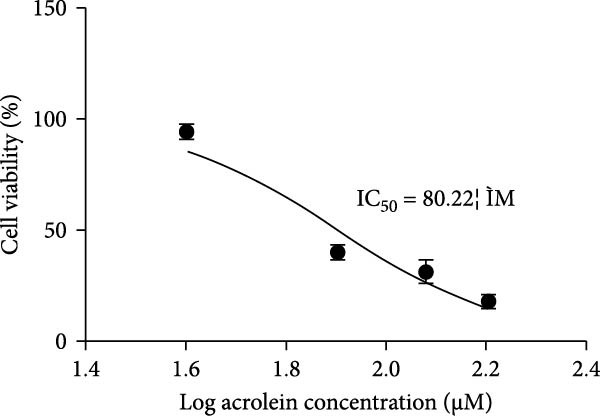
(B)

**Figure:**
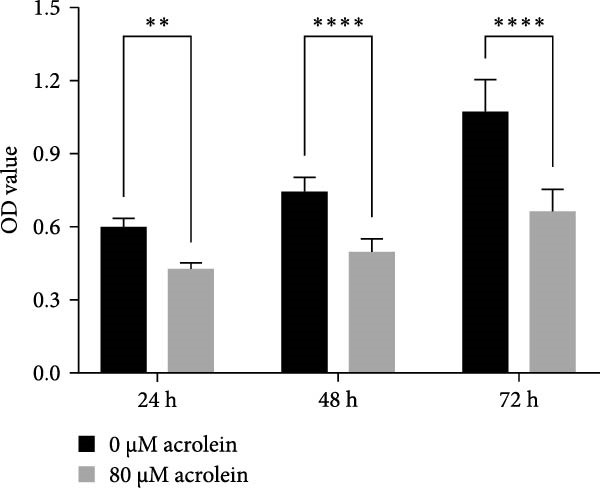
(C)

### 3.4. Effects of Acrolein on Colony Formation and Stemness Marker Expression in hOMECs

Acrolein treatment significantly impaired the colony forming of hOMECs, with the treated group showing a CFE of 4.533% compared to 11.8667% in the untreated group (*p* < 0.05). The expression of the stemness marker protein P63 was also markedly reduced in acrolein‐treated cells, being 25.7367% in the treated group versus 55.067% in the untreated group (*p* < 0.05). Further analysis using real‐time RT‐PCR revealed that the mRNA expression levels of P63 in acrolein‐treated cells were significantly decreased compared to untreated group (*p* < 0.01). These results indicate that acrolein exposure significantly diminishes the stem cell characteristics and viability of OMECs (Figure 4).

Figure 4Effect of acrolein on colony formation and stemness in human oral mucosal epithelial cells. (A (A1, A2)) Colony formation comparison between treated and control groups. (B (B1, B2)) P63 stem cell marker expression in the two groups, with red indicating P63 expression and blue indicating DAPI. (C) Colony‐forming efficiency (CFE) results. (D) Fluorescent expression of P63. (E) Relative expression of P63 mRNA. The acrolein‐treated group values are normalized by dividing by the P63 mRNA expression levels of the untreated control group.  ^∗∗^indicates *p*  < 0.01;  ^∗∗∗^indicates *p*  < 0.001.

**Figure:**
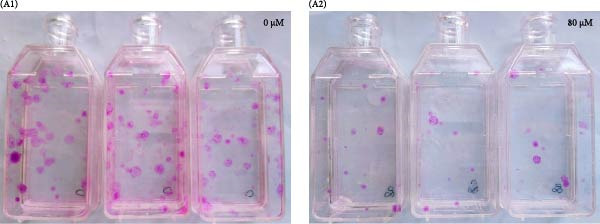
(A)

**Figure:**
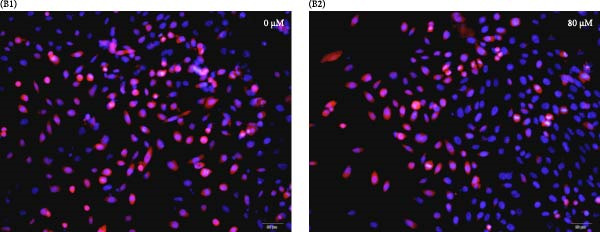
(B)

**Figure:**
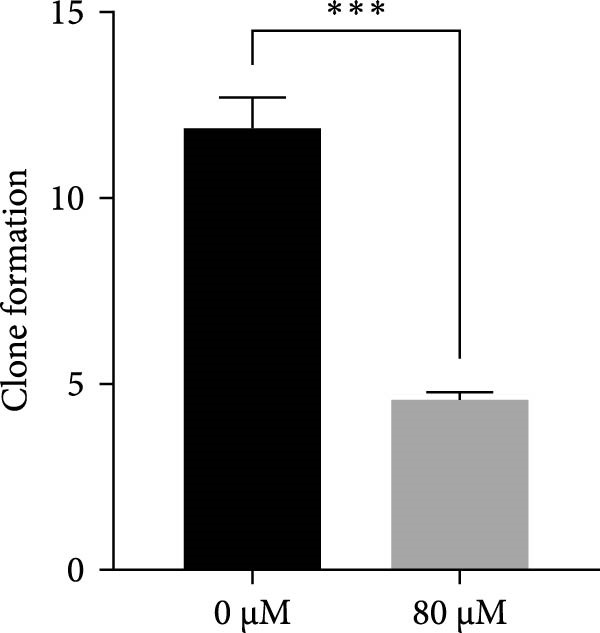
(C)

**Figure:**
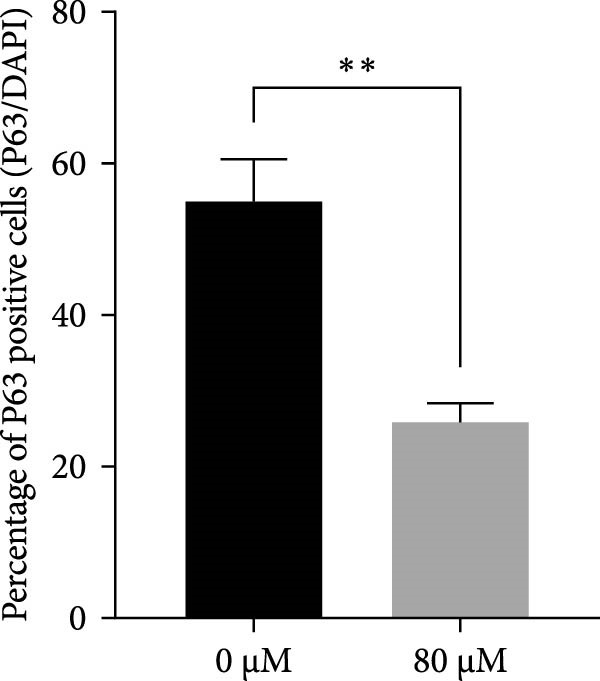
(D)

**Figure:**
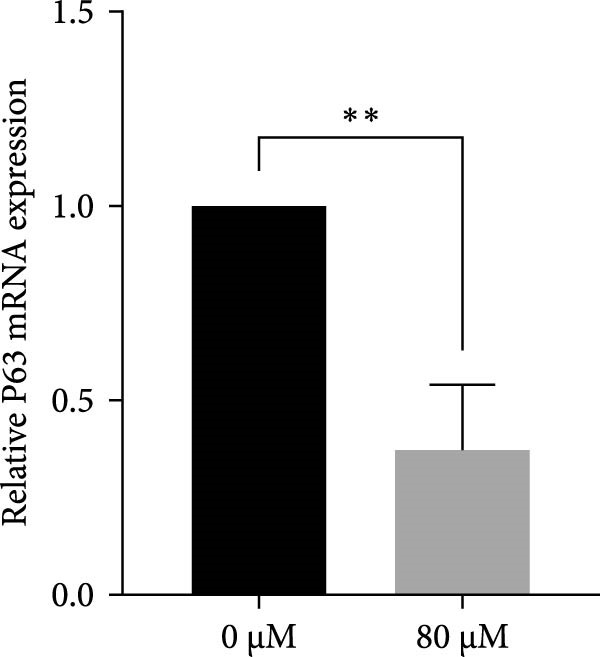
(E)

### 3.5. Effects of Acrolein on the Secretion of Repair Factors by hOMECs and Proliferation of Human Corneal Epithelial Cells

Acrolein treatment significantly reduced the secretion of crucial repair factors, specifically TGF‐β and HGF, however the release of bFGF without obviously change, by hOMECs (Figure 5C). This reduction in repair factor secretion was associated with impaired the wound healing potential of human corneal epithelial cells, as shown in Figure 5A,B. These findings parallel the therapeutic effects observed with oral mucosal epithelial sheet transplantation in LSCD patients. The data suggest that acrolein impairs the reparative function of OMECs by diminishing the secretion of key repair factors, potentially contributing to the reduced efficacy of cell‐based therapies in smokers.

Figure 5Effects of acrolein on secreted factors from human oral mucosal epithelial cells and their impact on human corneal epithelial cell wound healing. (A) Influence of condition medium derive from acrolein‐treated and normal‐condition from oral mucosal epithelial cells on corneal epithelial cell wound healing at different time points. (B) Statistical analysis of wound area across different time points for each group. (C) Comparison of repair factor concentrations in the conditioned media from each group.  ^∗^ indicates *p*  < 0.05;  ^∗∗^ indicates *p*  < 0.01.

**Figure:**
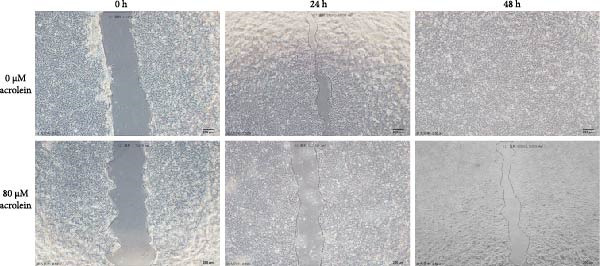
(A)

**Figure:**
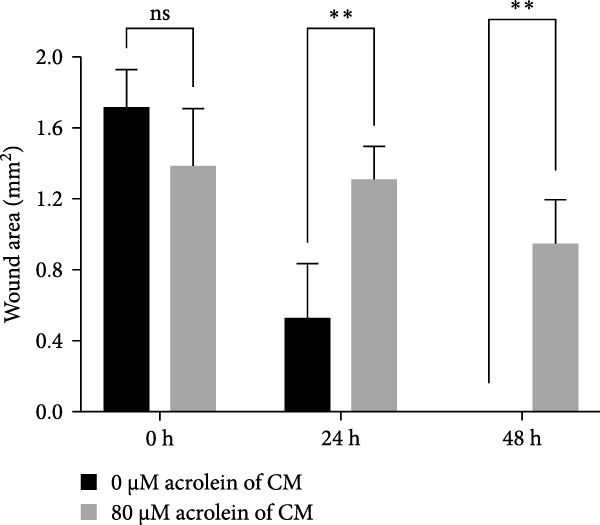
(B)

**Figure:**
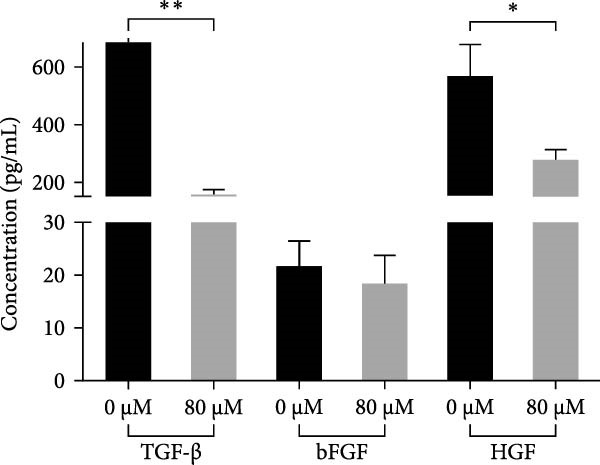
(C)

## 4. Discussion

In this study, we investigated the impact of smoking on the efficacy of autologous OMEC transplantation for treating LSCD and explored the effects of acrolein, a major component of cigarette smoke, on the biological properties of OMECs. Our findings indicate that smoking adversely affects treatment outcomes, as evidenced by earlier recurrence of symblepharon and less pronounced visual acuity improvements in the smoker group compared to the never smoker group. These observations align with previous studies that have highlighted the detrimental effects of smoking on various cellular and tissue repair processes [31].

This study is the first to report the effects of acrolein, a major component of cigarette smoke, on hOMECs in the context of LSCD treatment. Our in vitro experiments have demonstrated that acrolein exposure significantly impairs the CFE and reduces the expression of the stemness marker P63 in hOMECs. Additionally, acrolein diminished the secretion of critical repair factors such as TGF‐β and HGF, which are known to play crucial roles in promoting corneal epithelial cell repair. These factors have been well‐documented in the literature for their involvement in wound healing and tissue regeneration, suggesting that their reduced levels in acrolein‐treated cells could compromise the therapeutic efficacy of OMEC–based treatments in smokers [32, 33].

These results suggest that acrolein not only diminishes the regenerative potential of OMECs but also compromises their ability to support corneal epithelial repair. This novel insight highlights a possible mechanistic link between smoking‐related components and the reduced efficacy of cell‐based therapies for ocular surface diseases, emphasizing the need for further research into protective strategies against the detrimental effects of smoking.

Despite these significant results, the present study has certain limitations. First, the sample size was relatively small, which limits the statistical power of our findings and may affect the generalizability of the results. The small number of participants, particularly within subgroups such as smokers and never smokers, makes it challenging to draw definitive conclusions about the impact of smoking on treatment outcomes. Larger and multicenter studies are needed to confirm these observations and provide more robust data. Second, the diagnosis of total LSCD in this cohort was based primarily on clinical findings, including persistent epithelial defects, superficial vascularization, and loss of limbal architecture. Impression cytology or histologic confirmation was not feasible due to the severe ocular surface condition, which represents a limitation of the diagnostic assessment. Third, the study did not capture detailed smoking history, including duration, intensity, and cumulative exposure, which could influence the results [34]. These parameters could provide more precise correlations between smoking burden and treatment efficacy and should be incorporated into future studies.

Furthermore, while our in vitro analyses elucidated key cellular effects of acrolein, the underlying molecular pathways responsible for reduced stemness and paracrine function remain to be defined. Future mechanistic studies employing transcriptomic or proteomic profiling could help identify signaling pathways disrupted by acrolein exposure, thereby providing a foundation for developing targeted therapeutic interventions.

Finally, potential systemic confounders—such as diabetes mellitus [35], nutritional status [36], and other chronic conditions known to influence tissue repair [37]—were not fully controlled in this study. Accounting for these factors in future investigations will help isolate the specific contribution of smoking and enhance the interpretability of clinical outcomes. In addition, although smoking was associated with poorer ocular surface recovery in this cohort, the baseline imbalance in symblepharon severity between groups may also have contributed to differences in postoperative outcomes. Severe symblepharon represents extensive conjunctival fibrosis and mechanical restriction that may independently limit epithelial migration and ocular surface stability. Therefore, we cannot exclude the possibility that preoperative symblepharon severity acted as a confounding variable. Larger future studies should stratify outcomes based on symblepharon grade to determine the relative contributions of smoking vs. baseline structural severity to the success of autologous OMEC sheet transplantation.

## 5. Conclusion

Our study indicates that smoking, particularly exposure to acrolein, adversely impacts the efficacy of autologous OMEC transplantation for treating LSCD. Smoking was associated with reduced CFE, decreased P63 expression, and lower secretion of repair factors like TGF‐β and HGF, which are crucial for corneal epithelial repair.

These findings suggest that smoking‐related factors can diminish the regenerative potential of these cells. Future studies should validate these results with larger sample sizes and explore the underlying molecular mechanisms. This research could lead to improved strategies for enhancing the effectiveness of cell‐based therapies for LSCD, especially in patients who smoke.

## Disclosure

All authors reviewed the manuscript.

## Conflicts of Interest

The authors declare no conflicts of interest.

## Author Contributions

Zhuoshi Wang designed the research and analyzed the data. Mingqi Zhang and Le Wang performed the research and wrote this manuscript. Yuqiang Zhang and Hui Yu analyzed the data and provided feedback. Tao Yao perform surgical and observation.

## Funding

Research reported in this publication was supported by a grant from the Liaoning Province Joint Research Fund Project (Grant 2023‐MSLH‐074) and He’s University Project (Grant SJ202401)

## Data Availability

The data will be made available upon request.
